# The effect of replacing saturated fat with mostly n-6 polyunsaturated fat on coronary heart disease: a meta-analysis of randomised controlled trials

**DOI:** 10.1186/s12937-017-0254-5

**Published:** 2017-05-19

**Authors:** Steven Hamley

**Affiliations:** Faculty of Health, School of Exercise and Nutrition Sciences, Deakin University, 221 Burwood Highway, Burwood, 3125 Australia

**Keywords:** Saturated fat, Polyunsaturated fat, Omega 6, Diet heart hypothesis, Coronary heart disease, Clinical trial, Randomised controlled trial, Meta-analysis

## Abstract

**Background:**

A cornerstone of conventional dietary advice is the recommendation to replace saturated fatty acids (SFA) with mostly n-6 polyunsaturated fatty acids (PUFA) to reduce the risk of coronary heart disease (CHD). Many clinical trials aimed to test this advice and have had their results pooled in several meta-analyses. However, earlier meta-analyses did not sufficiently account for major confounding variables that were present in some of those trials. Therefore, the aim of the study was to account for the major confounding variables in the diet heart trials, and emphasise the results from those trials that most accurately test the effect of replacing SFA with mostly n-6 PUFA.

**Design:**

Clinical trials were identified from earlier meta-analyses. Relevant trials were categorised as ‘adequately controlled’ or ‘inadequately controlled’ depending on whether there were substantial dietary or non-dietary differences between the experimental and control groups that were not related to SFA or mostly n-6 PUFA intake, then were subject to different subgroup analyses.

**Results:**

When pooling results from only the adequately controlled trials there was no effect for major CHD events (RR = 1.06, CI = 0.86–1.31), total CHD events (RR = 1.02, CI = 0.84–1.23), CHD mortality (RR = 1.13, CI = 0.91–1.40) and total mortality (RR = 1.07, CI = 0.90–1.26). Whereas, the pooled results from all trials, including the inadequately controlled trials, suggested that replacing SFA with mostly n-6 PUFA would significantly reduce the risk of total CHD events (RR = 0.80, CI = 0.65–0.98, *P* = 0.03), but not major CHD events (RR = 0.87, CI = 0.70–1.07), CHD mortality (RR = 0.90, CI = 0.70–1.17) and total mortality (RR = 1.00, CI = 0.90–1.10).

**Conclusion:**

Available evidence from adequately controlled randomised controlled trials suggest replacing SFA with mostly n-6 PUFA is unlikely to reduce CHD events, CHD mortality or total mortality. The suggestion of benefits reported in earlier meta-analyses is due to the inclusion of inadequately controlled trials. These findings have implications for current dietary recommendations.

**Electronic supplementary material:**

The online version of this article (doi:10.1186/s12937-017-0254-5) contains supplementary material, which is available to authorized users.

## Introduction

A cornerstone of conventional dietary advice is the recommendation to reduce the intake of saturated fatty acids (SFA) as a means of reducing the risk of coronary heart disease (CHD). There are a few variations of this recommendation, these include: 1) advice to reduce the intake of SFA; 2) advice to replace SFA with monounsaturated fatty acids (MUFA) and mostly n-6 polyunsaturated fatty acids (PUFA); and 3) advice to replace SFA with mostly n-6 PUFA. Altogether, it is perhaps the single most influential recommendation in conventional dietary advice. It provides the basis to recommend low fat dairy and lean meats over full fat dairy and fattier cuts of meat; to recommend margarine and vegetable oils instead of butter and animal fats; and may lead to a greater emphasis on plant foods over animal foods. However, the evidence underlying this recommendation has been questioned by recent meta-analyses of observational studies and clinical trials [1–5].

*Fatty acids and plasma cholesterol*: the total concentration of plasma cholesterol (total-C) was one of the earliest risk factors identified for CHD and formed the basis of the lipid hypothesis, which is that reducing total-C would be expected to lower the risk of CHD [6]. A number of metabolic studies beginning in the 1950’s identified SFA and n-6 PUFA as major dietary influences of total-C [7]. This led to the development of the diet heart hypothesis, that decreasing SFA and/or increasing n-6 PUFA would be expected to lower the risk of CHD [8]. However, more recent evidence has identified the total-C:HDL-C ratio as being the measure of plasma cholesterol that is most predictive of CHD and is twice as predictive as total-C [9]. Therefore, the original lipid hypothesis and diet heart hypothesis should be modified to make predictions based on the total-C:HDL-C ratio, rather than total-C. When compared to carbohydrate, SFA does not significantly affect the total-C:HDL-C ratio as it raises both LDL-C and HDL-C [10], a point that is often ignored [11], although replacing SFA with either MUFA or PUFA would still lower the total-C:HDL-C ratio [10]. Consequently, as the fat in food is a mix of SFA, MUFA and PUFA, isocaloric substitution of carbohydrates with fat would be expected to reduce the total-C:HDL-C ratio [10].

*Evidence from observational studies*: meta-analyses of observational studies have consistently found that the intake of SFA is not independently associated with the incidence of CHD [1, 3, 12–16]. While some meta-analyses have found replacing SFA with PUFA is associated with a lower risk of CHD [12, 15], those results are not specific for SFA. Jakobsen et al. [12] found that replacing SFA with either MUFA or carbohydrate was not associated with a lower risk of CHD, while Farvid et al. [15] found that a higher intake of linoleic acid (18:2 n-6) was associated with a lower risk of CHD both independently and regardless of whether SFA or carbohydrate was replaced with linoleic acid. However, other meta-analyses of observational studies have not consistently found an inverse association between PUFA intake and CHD [3, 13, 14].

*Evidence from clinical trials*: there are several meta-analyses of clinical trials that aimed to test the diet heart hypothesis [3–5, 14, 17–20]. Half of these meta-analyses found a significant (*P* < 0.05) or near significant (*P* < 0.10) reduction in risk for CHD or cardiovascular disease (CVD) events when SFA was reduced or was replaced with mostly n-6 PUFA [14, 17–19]. Only Mozaffarian et al. [19] found a significant or near significant reduction in risk for CHD mortality, and only Skeaff & Miller [14] found a significant or near significant reduction in risk for total mortality (Table 1). The variation in results between the meta-analyses is partially due to differences in the clinical trials each of them included and, where relevant, how they were categorised, both of which are presented in Table 2. Despite slightly different aims, there is some consistency in the clinical trials included in these meta-analyses, with eight of the nineteen trials being included in a majority of them. Those eight trials [21–28], and another three [29–31], all involved replacing SFA with mostly n-6 PUFA. Those eleven trials will be referred to as the diet heart trials, with regard to the diet heart hypothesis, and are the focus of this study. The remaining trials included two with a Mediterranean diet intervention [32, 33] and six with a reduced fat intervention [34–39], neither of which strictly reduced SFA intake or replaced SFA with either carbohydrates, MUFA and/or PUFA, and each of which were only included in just one of the modified fat and reduced SFA meta-analyses respectively. As such, there is little evidence from clinical trials on the effect that reducing SFA in isolation, or replacing it with MUFA or carbohydrate, has on the risk of CHD or CVD outcomes, with the only trial to test either of these being the olive oil arm of the Rose Corn Oil Trial [21]. With disagreement between the meta-analyses on which trials to include, how they should be categorised and whether replacing SFA with mostly n-6 PUFA reduces CHD/CVD, closely examining the diet heart trials may help to resolve these issues.

**Table 1:** Results from earlier meta-analyses

Meta-analysis	Search criteria	CHD events	CHD mortality	Total mortality
Skeaff and Miller [14]	Altered PUFA/SFA ratio	0.83 (0.69-1.00)	0.84 (0.62-1.12)	0.88 (0.76-1.02)
		*P* = 0.050	*P* = 0.867	*P* = 0.083
Mozaffarian et al. [19]	Increase in total or n-6 PUFA	0.81 (0.70-0.95)	0.80 (0.65-0.98)	0.98 (0.89-1.08)
		*P* = 0.008	*P* < 0.05	
Hooper et al.^a,b^ [17]	Modified dietary fat	0.82 (0.66-1.02)	0.92 (0.73-1.15)	1.02 (0.88-1.18)
		*P* = 0.073	*P* = 0.46	*P* = 0.81
	Reduced and modified fat	0.77 (0.57-1.03)	0.98 (0.76-1.27)	0.97 (0.76-1.23)
		*P* = 0.077	*P* = 0.88	*P* = 0.78
Chowdhury et al.^c^ [3]	n-6 fatty acid supplementation	0.86 (0.69-1.07)	-	-
Schwingshackl and Hoffman^b^ [20]	PUFA vs. SFA in secondary prevention trials	0.93 (0.72-1.19)	1.05 (0.76-1.44)	0.99 (0.75-1.29)
		*P* = 0.54	*P* = 0.77	*P* = 0.91
Harcombe et al.^d^ [4]	Reduced or modified fat and published by 1983 or earlier	-	0.99 (0.78-1.25)	1.00 (0.87-1.15)
Hooper et al.^b^ [18]	Reduced SFA	0.83 (0.72-0.96)	0.95 (0.80-1.12)	0.97 (0.90-1.05)
		*P* = 0.013	*P* = 0.51	*P* = 0.47
Ramsden et al.^e^ [5]	Main analysis: replaced SFA with mainly n-6 PUFA	1.07 (0.80-1.41)	1.13 (0.83-1.54)	1.07 (0.90-1.27)
	Also includes dietary advice only or increased long chain n-3	-	1.00 (0.81-1.24)	1.00 (0.87-1.15)

Data are in relative risk (95% confidence interval). ^a^Hooper et al. (2012) categorised trials as replacing modified fat or modified and reduced fat, and performed a separate analysis for each category. ^b^Hooper et al. (2012), Schwingshackl & Hoffmann (2014), and Hooper et al. (2015) analysed CVD events and CVD mortality rather than CHD events and CHD mortality. ^c^Chowdhury et al. (2014) did not conduct an analysis for CHD mortality or total mortality. ^d^Harcombe et al. (2015) did not conduct an analysis for CHD events. ^e^Ramsden et al. (2016) included trials that replaced SFA with mainly n-6 PUFA in their main analysis and conducted a sensitivity analysis that included a further 3 trials that also increased intake of long chain n-3 PUFA in addition to replacing SFA with mainly n-6 PUFA or where participants were only provided with dietary advice

**Table 2:** The clinical trials included in the earlier meta-analyses

	Skeaff and Miller [14]	Mozaffarian et al. [19]	Hooper et al.^a,b^ [17]	Chowdhury et al. [3]	Schwingshackl and Hoffman [20]	Harcombe et al.^b^ [4]	Hooper et al.^b^ [18]	Ramsden et al.^c^ [5]
Rose Corn Oil Trial (RCOT) [21]	X		X (M)		X	X	X	X (MA)
Ball et al. [34]						X		
Oslo Diet Heart Study (ODHS) [22]	X	X	X (M)	X	X	X	X	X (SA)
National Diet Heart Study (NDHS) [29]			X (Both)					
Medical Research Council Trial (MRCT) [23]	X	X	X (M)	X	X	X	X	X (MA)
Los Angeles Veterans Administration Trial (LAVAT) [24]	X	X	X (M)	X		X	X	X (MA)
Finnish Mental Hospital Study (FMHS) [30]	X	X		X				
Sydney Diet Heart Study (SDHS) [25]			X (M)	X	X	X	X	X (MA)
Houtsmuller Diabetic Angiopathy Trial (HDAT) [31]			X (M)				X	
Minnesota Coronary Survey (MCS) [26]	X	X	X (M)	X				X (MA)
Diet and Reinfarction Trial (DART) [27]	X	X	X (M,R)	X	X		X	X (SA)
St Thomas Atherosclerosis Regression Study (STARS) [28]	X	X	X (M,R)	X	X		X	X (SA)
Black et al. [35]							X	
Moy et al. [36]							X	
Sondergaard et al. [32]			X (M,R)					
Ley et al. [37]							X	
Women’s Health Initiative (WHI) [38]							X	
Women’s Intervention Nutrition Study (WINS) [39]							X	
MeDiet [33]			X (M,R)					

^a^Hooper et al. (2012) categorised trials as either modified fat (M) or both modified and reduced fat (M,R) trials. NDHS included several experimental groups, some of which were prescribed a modified fat diet and others were prescribed a reduced and modified fat diet. Hooper et al. (2012) included these experimental groups individually and categorised them according to their dietary advice (Both). ^b^Hooper et al. (2012), Harcombe et al. (2015) and Hooper et al. (2015) included both the olive oil (MUFA) and the corn oil (n-6 PUFA) arms of RCOT as these meta-analyses examined the effect of fat modification. ^c^Ramsden et al. included trials that replaced SFA with mainly n-6 PUFA in their main analysis (MA) and conducted a sensitivity analysis (SA) that included trials that also increased intake of long chain n-3 PUFA in addition to replacing SFA with mainly n-6 PUFA (ODHS and STARS) or where participants were only provided with dietary advice (DART)

Upon inspection of the diet heart trials it is clear that many of them had substantial dietary or non-dietary differences between the intervention groups that were not related to SFA or mostly n-6 PUFA intake. The first indication of this is the categorisation of the diet heart trials by Hooper et al. [17] as either modified fat or both modified and reduced fat and by Ramsden et al. [5] as replacing SFA with mostly n-6 PUFA or also increasing long chain n-3 PUFA. But the differences in the diet heart trials go beyond reduced fat or higher long chain n-3 PUFA diets, and many of those differences have rarely been or yet to be acknowledged by the earlier meta-analyses.

*Trans fatty acids*: in some of the diet heart trials, only the experimental group (the high n-6 PUFA group) received advice to avoid major sources of industrial trans fatty acids (TFA), such as common/hard margarines, shortenings and/or hydrogenated oils [21, 22, 25, 28, 40, 41]. While in the other trials, the experimental group were provided with a lower amount of these foods compared to the control group (the high SFA group) [26, 29, 31, 42, 43] (Additional file 1). Therefore, in all the diet heart trials, the experimental group would be expected to have a lower intake of TFA compared to the control group. This was discussed by Ramsden et al. [2] in an earlier version of their meta-analysis, who described the diet heart trials as replacing both SFA and TFA with PUFA. TFA intake was only directly measured in STARS, where the experimental group had a much lower intake of TFA compared to the control group (1.08 vs. 1.80% of total energy intake) [44]. Ramsden et al. [2] estimated TFA intake in the control groups based on national food consumption data, but was only able to describe the TFA intake in most of the experimental groups as ‘restricted’. These estimations suggested most of the control groups had TFA intakes of approximately 1.5–2.5% of total energy intake, consistent with the control group in STARS, except that the control group in ODHS had an estimated TFA intake of 9.6% of total energy intake, due to the high use of hydrogenated marine oils in Norway at the time of the trial [2]. Due to the more detailed dietary information provided in FMHS [43], Ramsden et al. [2] was able to estimate TFA intake in both of the groups and found TFA intake to be lower in the experimental group in both hospital K (0.0 vs. 2.0% of total energy intake) and hospital N (0.2 vs. 0.6% of total energy intake). Ramsden et al. did not include NDHS or HDAT in any version of their meta-analysis or discuss TFA intake in either of those trials [2, 5, 45], but the diets provided to the control group in both NDHS and HDAT were most likely very high in TFA. Specifically, in NDHS, diet D in the first study and half the D diet groups in the second study were instructed to purchase ‘filled’ foods in which the fat was taken out and replaced with “either animal fat or hydrogenated shortening” [29]. Whereas in HDAT, the major source of fat for most of the participants in the control group was reported to be “saturated margarines” [31]. As the average cholesterol intake in the control group was identical to the experimental group and was 41% lower than the participants in the control group who ate butter, the ‘saturated margarines’ were not of animal origin and most likely comprised of hydrogenated vegetable oils [31]. The relative intake of TFA between the experimental group and control group in SDHS is controversial and less clear. The experimental group was advised to replace common margarines and shortenings with both liquid safflower oil and Miracle Margarine [45], which would be expected to reduce TFA intake. However, it is argued that the experimental group may have had a higher intake of TFA due to the use of Miracle Margarine, which has been suggested to have been rich in TFA at the time of the trial [46]. Therefore, it is possible that TFA intake in the experimental group was either higher, lower or similar to the control group. In response to Gutierrez [46], Ramsden et al. [47] argued that TFA intakes were likely similar between the groups based on the dietary differences briefly described above, the observed group differences in serum cholesterol and that adjusting for MUFA intake (an imperfect surrogate for trans fats as noted by Ramsden et al. [45]) did not have a noticeable effect on the results [47]. Differences in TFA intake between the experimental and control groups was not discussed by any of the other meta-analyses.

*Multifactorial dietary interventions*: ODHS and STARS both used a multifactorial dietary intervention, in which the dietary advice given to the experimental group included much more than just replacing SFA with mostly n-6 PUFA. Other dietary differences besides TFA intake included: 1) a higher intake of long chain n-3 PUFA (2.0% vs. usual intake (ODHS) [2] and 0.21 vs. 0.10% (STARS) [44]; 2) advice to consume more whole plant foods (ODHS [22] and STARS [44]); 3) advice to moderate sugar consumption and to increase fish and shellfish (ODHS) [22]; 4) sardines canned in cod liver oil that were supplied to the experimental group (ODHS) [22]; 5) advice to “avoid processed foods (eg, cookies, pastry, cakes)” (STARS) [28]; 6) advice to increase “plant-derived soluble fibre (chiefly pectin)” by 3.6 g/1000 kcal (STARS) [28]; and 7) a low calorie diet (1000–1200 kcal) that was prescribed for overweight participants (STARS) [28] (Additional file 1). Hooper et al. [17] judged ODHS and STARS as having a ‘high risk’ of bias related to being ‘free of dietary differences other than fat’, but included ODHS as a fat modification trial and STARS as a reduced and modified fat trial. Ramsden et al. discussed this issue in the 2010 and 2016 versions of their meta-analysis [2, 5], but included ODHS and STARS in a sensitivity analysis and categorised them as trials that increased both n-6 and long chain n-3 PUFA [5]. The use of a multifactorial dietary intervention in either ODHS or STARS was not discussed by any of the other meta-analyses [3, 4, 14, 18–20].

*Vitamin E*: in LAVAT, α-tocopherol intake in the control group was 9.4-fold lower than the experimental group (22.6 mg vs. 2.4 mg) [48] and only 16.0% of the current RDA (15 mg) [49]. Based on the average energy intake of the control group reported in the vitamin E paper (2400 kcal) [48] and the estimated energy intake (3150 kcal) and vitamin E (11.54 mg of α-tocopherol equivalents) per capita in the United States food supply between 1959–1968 [50], the vitamin E intake of the control group would be expected to be about 8.79 mg of α-tocopherol equivalents. This was not discussed by any of the meta-analyses.

*Cardiotoxic medication*: the control group in FMHS received more thioridazine in hospital N (0.82 vs. 1.79 average number of ‘normal doses’ per patient per day) and slightly less in hospital K (0.43 vs. 0.14), which averaged to an overall greater use in the control group (0.63 vs. 0.97) [43]. Ramsden et al. [2] discusses this issue and cites research that found thioridazine can cause electrocardiogram anomalies, which was the measure of CHD events in FMHS, and substantially increases the risk of sudden death. This was not discussed by any of the other meta-analyses.

An important aspect of randomised controlled trials is that the groups in the trial are treated identically except for the experimental treatment. This is to ensure that any differences between the groups in the outcome measures being tested are due to the experimental treatment and not due to another factor [51]. This can be challenging with dietary interventions but, at the very least, they should be free of the major differences that are mentioned in the previous paragraphs. These critical differences between the intervention groups have most likely substantially affected the results of those trials. The earlier meta-analyses either did not sufficiently acknowledge these issues or were simply not aware of such confounding factors. Therefore the aim of this meta-analysis is to account for the differences not related to SFA or mostly n-6 PUFA intake in the diet heart trials and to emphasise the results from those trials that most accurately test the effect of replacing SFA with mostly n-6 PUFA.

## Methods

I followed the PRISMA (www.prisma-statement.org) guidelines [52] throughout the design, implementation, analysis, and reporting of this meta-analysis.

### Literature search and eligibility criteria

A protocol for this meta-analysis has not been registered. The literature on clinical trials that examined the effect that replacing SFA with mostly n-6 PUFA has on CHD has already been thoroughly and recently searched by earlier meta-analyses [3–5, 14, 17–20], including two Cochrane meta-analyses by Hooper et al. [17, 18] that each contain a very comprehensive reference list. Clinical trials and their manuscripts were identified from these earlier meta-analyses. Trials were included if CHD events, CHD mortality or total mortality were reported, and if the trial involved replacing SFA with mostly n-6 PUFA. The latter was assessed on whether the trial had a control group and simultaneous decrease in SFA and increase in mostly n-6 PUFA of at least 20% in an experimental group, or if not reported, where the dietary advice provided strongly suggests that this occurred. Nineteen trials were identified (Table 2). Eleven trials were included (RCOT [21], ODHS [22], NDHS [29], MRCT [23, 40], LAVAT [24, 48, 53, 54], FMHS [30, 43, 55–58], SDHS [25, 45], HDAT [31], MCS [5, 26], DART [27, 41, 59] and STARS [28, 44]). RCOT, ODHS, MRCT and HDAT did not report SFA and PUFA intakes for both the experimental and control groups. However, the dietary advice provided to the experimental group in RCOT, ODHS and MRCT included comprehensive advice to substantially reduce SFA intake and very large doses of mostly n-6 PUFA rich oils to be taken daily [21, 22, 40], while the control group in HDAT was described as rich in SFA (“saturated fats 35 cal%”) and had a 4-fold lower intake of PUFA [31] (Additional file 1). Eight trials were excluded (Ball et al. [34], Black et al. [35], Moy et al. [36], Sondergaard et al. [32], Ley et al. [37], WHI [38], WINS [39] and MeDiet [33]), as the dietary information reported from each of these trials indicates that none had an intervention group that had a simultaneous decrease in SFA and increase in PUFA of at least 20% in an experimental group. Authors were contacted directly to request missing data or to clarify methods or results when necessary.

### Categorisation of the diet heart trials as ‘adequately controlled’ or ‘inadequately controlled’

As discussed in the introduction, there were many differences in the diet heart trials that were not related to SFA or mostly n-6 PUFA intake. In all of the diet heart trials the dietary advice or foods provided to participants would be expected to result in a lower TFA intake in the experimental group compared to the control group, particularly in ODHS, NDHS and HDAT. However, it is debated whether the experimental group in SDHS may have had a higher intake of TFA due to the use of a margarine that was potentially high in TFA. Furthermore, ODHS and STARS used a multifactorial dietary intervention, the control group in LAVAT had an insufficient vitamin E intake that was also 9.4-fold lower than the experimental group, and the control group in FMHS was prescribed more cardiotoxic medication on average. Therefore, to account for these differences, the clinical trials in this meta-analysis were categorised as ‘adequately controlled’ or ‘inadequately controlled’ and were subject to different subgroup analyses. Clinical trials categorised as adequately controlled are those that most accurately test the effect of replacing SFA with mostly n-6 PUFA, while the clinical trials categorised as inadequately controlled have too many dietary and/or non-dietary differences between the groups to be considered a valid test of replacing SFA with mostly n-6 PUFA. The clinical trials categorised as inadequately controlled include ODHS, NDHS, LAVAT, FMHS, HDAT, and STARS due to reasons discussed in the introduction and summarised above. The remaining trials, including RCOT, MRCT, SDHS, MCS, and DART, were categorised as adequately controlled. Due to debate over whether TFA intake in the SDHS experimental group was higher or lower than the control group, this trial was excluded in a sensitivity analysis of the adequately controlled trials.

### Calculating the risk ratio using person years where appropriate

MCS and FMHS reported their results as the number of events/deaths per 1000 person years, or per age-adjusted 1000 person years in the case of CHD mortality and total mortality in FMHS. Calculating the risk ratio (RR) using person years is important to do as the participants in those trials were patients in mental hospitals who could be discharged and readmitted, and any events/deaths that occurred during their absence would go unreported. The difference between calculating the RR using number of participants in each group rather than using person years is quite low in MCS [26], whereas in FMHS calculating the RR using the number of participants in each group often substantially underestimates the RR [30, 57]. The RevMan software automatically calculates the RR using the number of events and participants in each group that has been entered. Therefore the value entered for number of participants in each group has been altered to produce the correct RR when measured using person years or age-adjusted person years, but equal to the total number of participants in the trial so as to not affect the weighting of the trial. This was done by using the following equations. The equation for the RR using person years, where E is events/deaths and PY is person years, is: RR = (E_exp_∕PY_exp_)∕(E_con_∕PY_con_). To not affect the weighting, the total number of person years needs to equal the total number of participants: PY_exp_ + PY_con_ = N_exp_ + N_con_. Therefore: PY_exp_ = (N_exp_ + N_con_)∕(1 + (RR x E_con_∕E_exp_)); and PY_con_ = (N_exp_ + N_con_)∕(1 + (E_exp_∕(E_con_ x RR)))

### FMHS as an inadequately randomised trial

FMHS has been included in three earlier meta-analyses that are self-described as a meta-analysis of randomised controlled trials [3, 14, 19], but has been excluded by four for inadequate randomisation [2, 4, 17, 18] and its crossover design [4, 17, 18]. Participants were allocated by hospital and were not individually randomised in FMHS, and while it has been suggested to be a cluster randomised trial [19], there would only have been 2 clusters and there is actually no mention of random allocation of the hospitals in the publications from the trial [30, 43, 55–58]. The purpose of randomisation is to ensure that there as few differences between the groups at baseline as possible [51] and, in this respect, FMHS appears to be inadequately randomised. There were a number of confounding variables, including minor differences in baseline characteristics such as age, BMI, smoking and blood pressure, as well as the critical difference in cardiotoxic medication use [43, 57]. In addition, the fact that on average the participants in the control group remained in the hospitals longer than those in the experimental group, which led to an overestimation of the effect size (see above), also points to inadequate randomisation or differences in treatment. Therefore, FMHS was excluded in a separate analysis that only includes adequately randomised trials.

### Statistical analysis

For each outcome measure, a random-effects inverse-variance meta-analysis was performed to calculate the RR for: 1) the overall pooled effect for all trials; 2) the adequately randomised trials (which excluded FMHS); 3) the adequately controlled trials; 4) the inadequately controlled trials; and 5) the adequately controlled trials where SDHS was excluded in a sensitivity analysis. All statistical tests were 2-sided and significance was set at *P* < 0.05. Heterogeneity was assessed using the I^2^ test, and considered significant where I^2^ > 50% [60]. The potential of publication bias was assessed by visual inspection of funnel plots. All data were analysed using the REVIEW MANAGER V.5.1 software, provided by the Cochrane Collaboration (http://ims.cochrane.org/revman).

## Results

Characteristics of the diet heart trials are outlined in Table 3. Many of the diet heart trials only included males with pre-existing CHD. Only FMHS, HDAT and MCS included both men and women, NDHS and HDAT included participants without pre-existing CHD, and LAVAT, FMHS, MCS included participants both with and without pre-existing CHD. All the trials used a parallel design, except FMHS, which used a crossover design.

**Table 3:** Characteristics of the diet heart trials

	Allocation	Blinding	Design	Sex	Population	Prevention	Number of participants	Age on entry	Follow up (Years)	Years of the trial
RCOT [21]	Random	Single	Parallel	Not reported	Free Living	Secondary	54	<70	2.0	Not reported
ODHS [22]	Random	Single	Parallel	Male	Free Living	Secondary	412	30-64	5.0	1958-1963
NDHS [29]	Random	Single & Double	Parallel	Male	Free Living	Primary	2032	45-55	1.0	1962-1964
MRCT [23]	Random	Single	Parallel	Male	Free Living	Secondary	393	<60	2.0-7.0	1960-1967
LAVAT [24]	Random	Double	Parallel	Male	Domiciliary	Both	846	≥55	8.0	1959-1968
FMHS [30, 43, 57]^a^	Assigned by Hospital	Single	Crossover	Both	Mental Hospital	Primary Both	1635^b^ 10612	34-64/44-64 >15	6.0	1959-1971
SDHS [25]	Random	Single	Parallel	Male	Free Living	Secondary	458	30-59	2.0-7.0	1966-1973
HDAT [31]	Random	Not reported	Parallel	Both	Free Living	Primary	102	Not reported	5.0	1973-1978
MCS [26]	Random	Double	Parallel	Both	Mental Hospital	Both	9057	<30 to >70	4.5	1968-1973
DART [27, 59]	Random	Single	Parallel	Male	Free Living	Secondary	2033	30-69	2.0	1983-1989
STARS [28]	Random	Single	Parallel	Male	Free Living	Secondary	55	<66	3.25	1987-1991^c^

^a^Data for CHD events in FMHS comes from male and female patients aged 34–64 and 44–64 respectively and “whose initial electrocardiogram was free from coronary patterns”, whereas data for mortality comes from all patients aged >15. ^b^For an unknown reason, there were five fewer participants for total CHD events compared to major CHD events [57]. ^c^G Watts, personal communication, April 28, 2016

Dietary information is presented in Additional file 1. There is a substantial difference in the reported intakes of SFA and PUFA between the experimental and control groups (Table 4), indicating a high level of compliance. The only exception is STARS, where PUFA intake differed by only 2.6% of total energy intake, reflective of the more modest PUFA target in STARS (8% of total energy intake) [28]. The relatively high level of compliance in the diet heart trials is supported by the consistent reductions in total-C in the experimental group at follow up, which occurred in all the diet heart trials except DART and HDAT, often with minimal change in the control group (Table 5).

**Table 4:** Saturated fat and polyunsaturated fat intake in the diet heart trials

	Experimental group			Control group		
	SFA (%)	PUFA (%)	P:S	SFA (%)	PUFA (%)	P:S
RCOT [21]^a^						
ODHS [22]^b^	8.5	20.6	2.44			
NDHS [29]^c^	7.7	11.1	1.48	12.0	5.0	0.41
MRCT [23]^d^			2.00			0.17
LAVAT [53]	9.2	15.6	1.70	16.4	4.9	0.30
FMHS [43]	8.6	12.7	1.48	17.2	4.3	0.25
SDHS [25]	9.8	15.1	1.70	13.5	8.9	0.80
HDAT [31]		18.4			4.8	
MCS [26]	9.2	14.7	1.60	18.3	5.2	0.28
DART [41]	11.2	9.5	0.85	14.9	6.7	0.45
STARS [44]	8.9	7.3	0.90	17.1	4.7	0.30

*Abbreviations*: *SFA* (%) the percentage of total energy intake from saturated fatty acids, *PUFA* (%) the percentage of total energy intake from polyunsaturated fatty acids, *P:S* the ratio of polyunsaturated fatty acid intake to saturated fatty acid intake

^a^RCOT did not report either SFA or PUFA intake or the P:S ratio. However, the corn oil group reported consuming an average of 64 g of corn oil and 2070 kcal per day [21], so the corn oil alone would provide approximately 35.0 g of PUFA [88] or 15.2% of total energy intake from PUFA [21]. ^b^ODHS only reported data on dietary intakes from 17 “especially conscientious” participants in the experimental group and from none of the participants in the control group [22]. ^c^The values for NDHS come from a weighted average of the experimental and control groups respectively. ^d^MRCT did not report SFA or PUFA intake for either group. However, the experimental group reported consuming an average of 80 g of soybean oil and 2380 kcal per day, so the soybean oil alone would provide approximately 46.2 g of PUFA [88] or 17.5% of total energy intake from PUFA

**Table 5:** Plasma cholesterol in the diet heart trials

	Experimental group			Control group		
	Baseline (mg/dl)	Follow up (mg/dl)	Change (mg/dl)	Baseline (mg/dl)	Follow up (mg/dl)	Change (mg/dl)
RCOT [21]			−20			−3
ODHS [22]	296	244	−52	296	285	−11
NDHS [29]^a^	232	208	−24	229	224	−5
MRCT [23]	272	239	−33	273	269	−4
LAVAT [54]	233	190	−43	234	201	−33
FMHS [43, 57]^b^		231			270	
SDHS [25]	281	250	−31	282	262	−20
HDAT [31]^d^	263	249	−14	267	267	0
MCS [26]	205	175	−30	204	203	−1
DART [27]	250	243	−7	250	253	+3
STARS [28]	278	239	−39	273	268	−5

^a^The values for NDHS come from a weighted average of the experimental and control groups respectively. ^b^Due to the crossover design used in FMHS, only the values for total cholesterol at the end of each diet period are presented in this table. ^c^The actual numbers for total plasma cholesterol were not reported in HDAT and the numbers in this table were estimated from graphs reported in the study. This estimation is consistent with Hooper et al. [18], as they estimated from the graph that the average plasma cholesterol of the experimental group was 18 mg/dl lower than the control group at the end of the trial

### Major CHD events

**Fig. 1:**
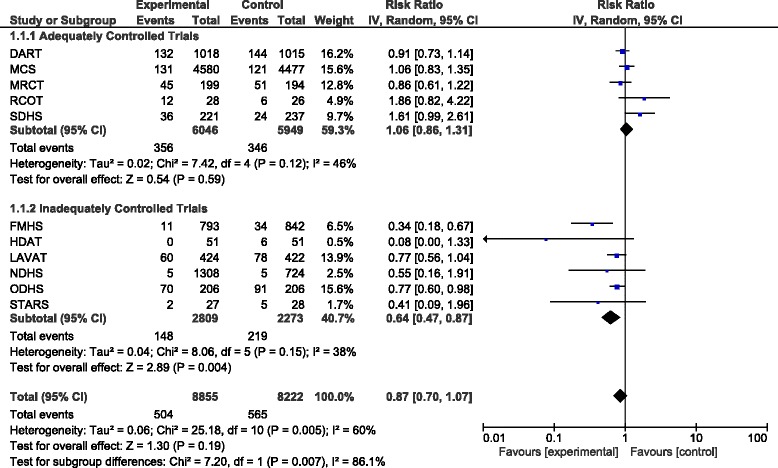
Forest plot showing pooled RR with 95% CI for the number of major CHD events

When pooling the results of all trials together there was a total of 1069 major CHD events (includes myocardial infarction and sudden death) in 17077 participants. The total pooled RR was 0.87 (95% CI 0.70–1.07, *P* = 0.19). Exclusion of FMHS as an inadequately randomised trial increased the pooled RR to 0.93 (95% CI 0.77–1.11, *P* = 0.40). When only pooling results from the adequately controlled trials the pooled RR was 1.06 (95% CI 0.86–1.31, *P* = 0.59) and excluding SDHS from this subgroup in a sensitivity analysis decreased the pooled RR to 0.98 (95% CI 0.83–1.16, *P* = 0.80). The results of the adequately controlled trials and the inadequately controlled trials as subgroups were significantly different (*P* = 0.007) and there was evidence of significant heterogeneity (I^2^ = 60%; Fig. 1).

### Total CHD events

**Fig. 2:**
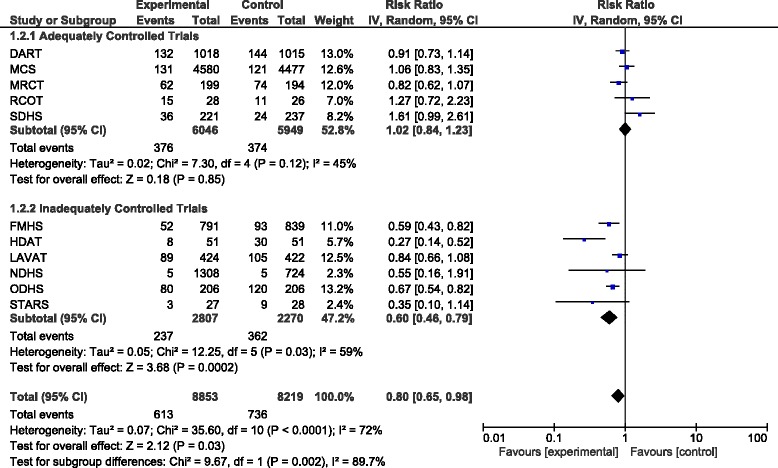
Forest plot showing pooled RR with 95% CI for the number of total CHD events

When pooling the results of all trials together there was a total of 1349 CHD events (also includes soft CHD events such as angina) in 17072 participants. The total pooled RR was 0.80 (95% CI 0.65–0.98, *P* = 0.03). Exclusion of FMHS as an inadequately randomised trial increased the pooled RR to 0.83 (95% CI 0.67–1.03, *P* = 0.10). When only pooling results from the adequately controlled trials the pooled RR was 1.02 (95% CI 0.84–1.23, *P* = 0.85) and excluding SDHS from this subgroup in a sensitivity analysis decreased the pooled RR to 0.95 (95% CI 0.83–1.09, *P* = 0.45). The results of the adequately controlled trials and the inadequately controlled trials as subgroups were significantly different (*P* = 0.002) and there was evidence of significant heterogeneity (I^2^ = 72%; Fig. 2).

### CHD Mortality

**Fig. 3:**
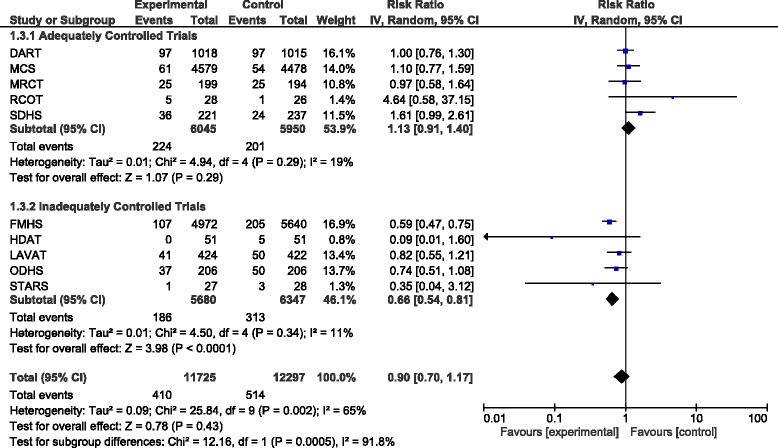
Forest plot showing pooled RR with 95% CI for CHD mortality

When pooling the results of all trials together there was a total of 924 deaths due to CHD in 24022 participants. The total pooled RR was 0.90 (95% CI 0.70–1.17, *P* = 0.43). Exclusion of FMHS as an inadequately randomised trial increased the pooled RR to 0.98 (95% CI 0.79–1.23, *P* = 0.88). When only pooling results from the adequately controlled trials the pooled RR was 1.13 (95% CI 0.91–1.40, *P* = 0.29) and excluding SDHS from this subgroup in a sensitivity analysis decreased the pooled RR to 1.04 (95% CI 0.85–1.27, *P* = 0.71). The results of the adequately controlled trials and the inadequately controlled trials as subgroups were significantly different (*P* = 0.0005) and there was evidence of significant heterogeneity (I^2^ = 65%; Fig. 3).

### Total mortality

**Fig. 4:**
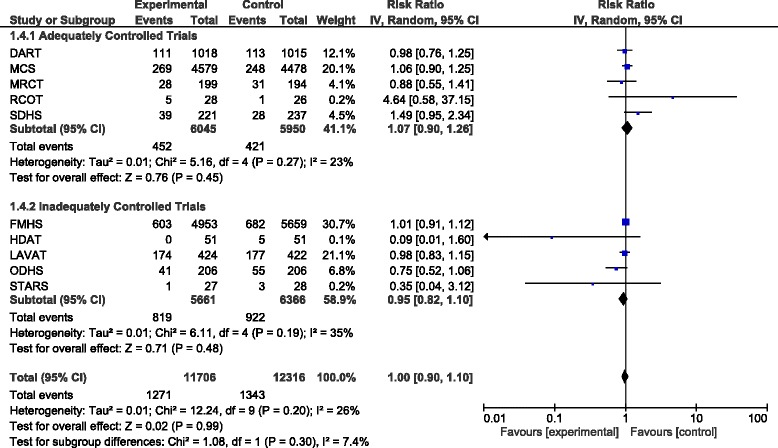
Forest plot showing pooled RR with 95% CI for total mortality

When pooling the results of all trials together there was a total of 2614 deaths in 24022 participants. The total pooled RR was 1.00 (95% CI 0.90–1.10, *P* = 0.99). Exclusion of FMHS as an inadequately randomised trial did not alter the RR (RR = 0.99; 95% CI 0.86–1.15, *P* = 0.91). When only pooling results from the adequately controlled trials the pooled RR was 1.07 (95% CI 0.90–1.26, *P* = 0.45) and excluding SDHS from this subgroup in a sensitivity analysis decreased the pooled RR to 1.03 (95% CI 0.90–1.17, *P* = 0.69). The results of the adequately controlled trials and the inadequately controlled trials as subgroups were not significantly different (*P* = 0.30) and there was no evidence of significant heterogeneity (I^2^ = 26%; Fig. 4).

A summary of the results is presented in Table 6.

**Table 6:** A summary of the results

	All trials	All trials excluding FMHS	Adequately controlled trials	Adequately controlled trials excluding SDHS	Inadequately controlled trials
Major CHD Events	0.87 (0.70-1.07)	0.93 (0.77-1.11)	1.06 (0.86-1.31)	0.98 (0.83-1.16)	0.64 (0.47-0.87)
	*P* = 0.19	*P* = 0.40	P = 0.59	*P* = 0.80	*P* = 0.004
	I^2^ = 60%	I^2^ = 48%	I^2^ = 46%	I^2^ = 17%	I^2^ = 38%
Total CHD Events	0.80 (0.65-0.98)	0.83 (0.67-1.03)	1.02 (0.84-1.23)	0.95 (0.83-1.09)	0.60 (0.46-0.79)
	*P* = 0.03	*P* = 0.10	P = 0.85	*P* = 0.45	*P* = 0.0002
	I^2^ = 72%	I^2^ = 71%	I^2^ = 45%	I^2^ = 1%	I^2^ = 59%
CHD Mortality	0.90 (0.70-1.17)	0.98 (0.79-1.23)	1.13 (0.91-1.40)	1.04 (0.85-1.27)	0.66 (0.54-0.81)
	*P* = 0.43	*P* = 0.88	P = 0.29	*P* = 0.71	*P* < 0.0001
	I^2^ = 65%	I^2^ = 39%	I^2^ = 19%	I^2^ = 0%	I^2^ = 11%
Total Mortality	1.00 (0.90-1.10)	0.99 (0.86-1.15)	1.07 (0.90-1.26)	1.03 (0.90-1.17)	0.95 (0.82-1.10)
	*P* = 0.99	*P* = 0.91	P = 0.45	*P* = 0.69	*P* = 0.48
	I^2^ = 26%	I^2^ = 34%	I^2^ = 23%	I^2^ = 0%	I^2^ = 35%

Data are in relative risk and then 95% confidence intervals in parentheses, with *P* values and I^2^ values below

### Publication bias

Visual inspection of the funnel plots (Additional file 2, Additional file 3, Additional file 4, Additional file 5: Figure S4) shows a fairly symmetric distribution. There was some asymmetry for the funnel plots for major CHD events (Additional file 2), suggesting the possibility that some small studies with more major CHD events in the experimental group may be missing from this review. Analysis for publication bias is limited by some inconsistency in the funnel plots and the small number of studies included (*N* = 11), and may simply reflect methodological differences rather than publication bias. However, it should be noted that CHD and CVD mortality were not reported in the original publication for SDHS [25, 45] and that it took approximately 16 years from the end of the trial (1973) for MCS to be published in a peer-reviewed article in 1989 [26]. Both SDHS and MCS were unfavourable trials for the popular diet heart hypothesis.

## Discussion

Available evidence from adequately controlled randomised controlled trials suggest replacing SFA with mostly n-6 PUFA is unlikely to reduce CHD events, CHD mortality or total mortality. When the results of all eleven trials are pooled together it appears that replacing SFA with mostly n-6 PUFA significantly reduces total CHD events, but not major CHD events, CHD mortality or total mortality. However, those analyses include results from inadequately randomised trials and inadequately controlled trials. Excluding FMHS as an inadequately randomised trial increases the pooled RR towards 1.00 for all outcomes except total mortality and the reduced risk for total CHD events loses statistical significance. Excluding the inadequately controlled trials and just pooling results from the adequately controlled trials, shows no significant effect on any outcome measure, whether SDHS is excluded in the sensitivity analysis or not. As the adequately controlled trials most accurately test the effect of replacing SFA with mostly n-6 PUFA, the results of this meta-analysis suggest that replacing SFA with mostly n-6 PUFA is unlikely to have either a beneficial or an adverse effect on CHD events, CHD mortality and total mortality.

A novel approach of this meta-analysis was identifying the diet heart trials with substantial confounding variables and then excluding them from the final analysis, thereby obtaining results from those trials that most accurately test the effect of replacing SFA with mostly n-6 PUFA. This was achieved by categorising the trials as adequately controlled or inadequately controlled depending on whether there were substantial dietary or non-dietary differences between the experimental and control groups that were not related to SFA or mostly n-6 PUFA intake, and then perform a separate subgroup analysis for each category. Limitations of this method include that the categorisation is difficult to be based on criteria developed prior to the literature review, and that it is an estimation of the effects of the confounding variables in the trials. However, ODHS, LAVAT, FMHS and STARS had clear evidence of substantial differences between the experimental and control groups that were not related to SFA or mostly n-6 PUFA intake as discussed in the introduction. In addition, the results add some support to this method. There was significant heterogeneity for major CHD events, total CHD events and CHD mortality, indicating a strong likelihood of there being methodological differences between the diet heart trials. Furthermore, there was a significant difference between the two subgroups for major CHD events, total CHD events and CHD mortality. Another limitation of this method is that NDHS and HDAT were classified as inadequately controlled as the control groups in those trials most likely had a substantially higher TFA intake than the experimental groups, but this is not certain. However, the results add some support to the categorisation of these trials as inadequately controlled as well. Notably, NDHS had a stronger effect size than the pooled result of the inadequately controlled trials and HDAT had a stronger effect size than all the other inadequately controlled trials. These results add concern that those trials were indeed confounded by substantial differences in TFA intake.

There is debate over whether TFA intake in the SDHS experimental group was higher or lower than the control group. Therefore, the second method was to exclude SDHS in a sensitivity analysis of the adequately controlled trials. The results add some support to this method as well. SDHS was an outlier in the adequately controlled trials and responsible for most of the heterogeneity in this subgroup, suggesting that TFA intake may have been higher in the experimental group of SDHS. However, this could alternatively be explained by the other adequately controlled trials being confounded by small differences in TFA intake and other small dietary differences, in combination with the explanations by Ramsden et al. that TFA is unlikely to been a major factor in SDHS [45, 47]. The experimental groups of the adequately controlled trials were instructed to avoid “fried foods, pastry and cakes (except plain sponge)” (RCOT) [21]; “other margarines, cooking fat, other oils and most biscuits and cakes” (MRCT) [23]; and to limit other sources of fat “(e.g. cakes, pastries, biscuits, meat pies and pasties, crisps, chocolates and toffees)” to four portions per week with at least two to be made with a polyunsaturated fat (DART) [41]; and in the case of MCS, using corn oil in place of the usual hospital cooking fats that included hydrogenated oils, and from the control group receiving common margarines and shortenings [5]. Therefore, the actual RR of replacing SFA with mostly n-6 PUFA may be higher than what is reported in this study had TFA intake been better controlled for in those trials, and excluding SDHS in the sensitivity analysis may have been inappropriate.

*CHD events and mortality*: half the earlier meta-analyses reported a significant or near significant reduction in risk for CHD/CVD events [14, 17–19], and almost all the earlier meta-analyses did not find a significant reduction in risk for CHD/CVD mortality [3–5, 14, 17, 18, 20]. When pooling the results of all trials together this meta-analysis obtained a similar result, with a significant reduction in risk for total CHD events, but not for major CHD events and CHD mortality. When only pooling results from the adequately controlled trials, the RR for both CHD events and CHD mortality was higher than most other meta-analyses due to the exclusion of the inadequately controlled trials, but similar to the main analysis by Ramsden et al. [5]. Therefore, the suggestion of benefits reported in most earlier meta-analyses is due to the inclusion of inadequately controlled trials.

Mozaffarian et al. [19] was the only meta-analysis to find a significant reduction in risk for CHD mortality, which is mostly due to their inclusion of FMHS and their exclusion of SDHS. Skeaff and Miller [14] was the only other meta-analysis that included FMHS for CHD mortality and excluded SDHS. However, Skeaff and Miller [14] did not find a significant reduction in risk for CHD mortality. This is most likely because their values for CHD mortality came from the small subgroup of participants for assessing CHD events. Therefore, those values were much lower than the values for all CHD mortality in the trial and this substantially lowered the weighting of FMHS in their meta-analysis.

*Total mortality*: this meta-analysis found no effect for total mortality regardless of whether FMHS or all the inadequately controlled trials were excluded. This is consistent with almost all the earlier meta-analyses [3–5, 17–20]. Skeaff and Miller [14] is the only meta-analysis that found a significant or near-significant reduction in risk for total mortality. Despite including the same trials and using similar figures as Mozaffarian et al. [19] for total mortality, Skeaff and Miller [14] obtained a near-significant result for total mortality, whereas Mozaffarian et al. [19] found no effect. This is most likely because Skeaff and Miller [14] calculated the RR for FMHS using the number of participants in each group, whereas Mozaffarian et al. [19] calculated the RR for total mortality in FMHS using age-adjusted person years for women, and obtained an RR similar to non-age-adjusted person years for men. This had a large impact on the results, as FMHS contributed 36.27% of the weighting in the meta-analysis by Skeaff & Miller [14], and in FMHS, calculating the RR for total mortality using the number of participants in each group rather than using age-adjusted person years underestimates the RR by 17.9% in men and 33.9% in women.

Replacing SFA with PUFA reduces the total-C:HDL-C ratio [10], which appears to be the most predictive blood cholesterol risk factor for CHD [9]. In addition, some meta-analyses of observational studies [12, 15], but not all [3, 13, 14], have found an inverse association between PUFA intake and the incidence of CHD. Therefore, it could be hypothesised that replacing SFA with PUFA would reduce CHD. However, this hypothesis is refuted by the currently available evidence from randomised controlled trials, which are higher on evidence hierarchies than observational studies (while risk factors and mechanisms such as cholesterol are not included on these evidence hierarchies) and are the gold standard in evidence-based medicine [61]. While clinical trials are not perfect, this discordant result is most likely due to the limitations inherent in observational studies and in the use of risk factors or proposed mechanisms.

Observational studies are not randomised, and therefore it can be quite likely for there to be other differences between groups of people besides the specific diet or lifestyle aspect that is being measured. These other differences may substantially affect the results and are referred to as confounding variables [62, 63]. While many observational studies attempt to control for a number of confounding variables, this does not sufficiently work all the time as was the case with hormone replacement therapy for CVD [64], antioxidant vitamin supplementation for CVD [65, 66] and dietary fibre supplementation for colorectal cancer [67]. It is possible that confounding variables also explain the discordant result between observational studies and clinical trials regarding the replacement of SFA with PUFA for CHD. SFA intake is associated with behaviours indicating lower health consciousness [68–70], whereas PUFA intake is either associated with behaviours indicating greater health consciousness [71] or does not appear to be related to health consciousness [69]. In addition, a meta-analysis found that smokers have a significantly lower intake of PUFA [72].

Replacing SFA with PUFA reduces the total-C:HDL-C ratio, and a higher total-C:HDL-C is associated with a greater risk of CHD. Therefore, it could be claimed that replacing SFA with PUFA will reduce the risk of CHD. However, this assumes that replacing SFA with PUFA only affects the total-C:HDL-C ratio and/or that the total-C:HDL-C ratio is the only factor in the development of CHD. Despite reducing the total-C:HDL-C ratio, replacing SFA with mostly n-6 PUFA does not appear to affect the incidence of CHD or CHD mortality in randomised controlled trials. This suggests that the larger risk associated with a higher total-C:HDL-C ratio is mediated through other environmental and/or genetic factors; and that the likely beneficial effect that replacing SFA with mostly n-6 PUFA has on the total-C:HDL-C ratio may be counterbalanced by other mechanisms, such as higher n-6 PUFA intake increasing LDL oxidation [73–75]. In addition to the three examples mentioned above, there are further examples where targeting risk factors or proposed mechanisms have yielded unexpected results. These include the use cholesterol ester transfer protein inhibitors to reduce the total-C:HDL-C ratio for CHD [76]; vitamin B6, B9 and B12 supplementation to lower homocysteine for CHD [77, 78]; and that carnitine reduces CHD events and total mortality [79] even though it increases trimethylamine N-oxide, which is associated with a higher risk of CHD [80].

Similarly, the methods used to alter nutrient intakes can also influence the results of a trial. The diet heart trials used a number of methods to reduce SFA intake, including advice to limit consumption of fatty meats and full fat dairy, and advice to reduce ‘commercial baked goods’ or ‘cakes and biscuits’ (Additional file 1); and some of these methods would be expected produce more or less favourable results than others. This is well illustrated by some contradictory clinical trials investigating the effect that increasing SFA intake has on endothelial function, where the difference between these trials is most likely due to what foods were used to represent SFA, other fatty acids and carbohydrate [81]. The first trial found that SFA impairs endothelial function, but compared butter (SFA) to almonds and high MUFA margarine (MUFA), walnuts and high PUFA margarine (PUFA), and sultanas and jam/marmalade (high glycemic load) [82]. This made the first trial confounded by the extra protein, fibre, micronutrients and phytonutrients that are naturally present in whole foods such as almonds, walnuts and sultanas [81]. Whereas the second and third trials had far more balanced interventions regarding food quality and found that SFA does not impair endothelial function [81, 83]. In the case of the first trial, the cumulative effect from differences in all the other nutrients and chemicals found in whole foods most likely influenced the results, and are likely to be more influential than the fatty acids and carbohydrates that are being intentionally manipulated. As such, there are likely to be issues in generalising the effect of a nutrient to foods rich in that nutrient and vice versa. This has important implications for current conventional dietary advice, which tends to be nutrient-based rather than food-based.

There are a few other limitations of this study. The adequately controlled trials are those that most accurately test the effects of replacing SFA with mostly n-6 PUFA. However, this subgroup only includes five trials, or four trials when SDHS is excluded in the sensitivity analysis, and so this meta-analysis is limited by a small number of appropriate randomised controlled trials. In addition, the participants in the experimental groups of the diet heart trials often reported a very high intake of PUFA. The average intake of PUFA across all trials was at least 14.3% of total energy intake, and this extreme dietary shift helped the participants achieve solid reductions in plasma cholesterol. Almost all the experimental groups had average PUFA intakes above 10% of total energy intake, except for DART (9.5%) and STARS (7.3%), and this exceeds current recommendations from a number of health bodies such as the American Heart Association (5–10%) [84], the Institute of Medicine (5–10%) [85] and the Academy of Nutrition and Dietetics (3-10%) [86]. However, even these recommendations, and current intakes (~7.21%), are high compared to the average n-6 PUFA intake in the United States at the beginning of the 20th century (2.79%), before the widespread use of modern vegetable oils [87]. In light of this modern shift in n-6 PUFA intake, it is important to test these recommendations against historical intakes using high quality randomised controlled trials. Lastly, the method to raise mostly n-6 PUFA intake in the diet heart trials relied heavily on vegetable oils: either in ‘filled foods’ [26, 29, 43, 53], using them in place of other added fats [22, 23, 25, 31, 41, 43], and/or prescribing daily doses as a form of nutritional supplementation [21–23]. Therefore, these results should not be generalised to other foods high in mostly n-6 PUFA such as nuts and seeds.

## Conclusion

In conclusion, available evidence from adequately controlled randomised controlled trials suggest replacing SFA with mostly n-6 PUFA is unlikely to reduce CHD events, CHD mortality or total mortality. The suggestion of benefits reported in earlier meta-analyses is due to the inclusion of inadequately controlled trials. This has implications for current dietary advice where recommendations to reduce SFA and/or replace SFA with mostly n-6 PUFA feature prominently, as maintaining these recommendations is unlikely to have the intended effect and may reduce efforts to get people to adopt other lifestyle changes that are more likely to be beneficial.

## Additional files

Additional file 1: The dietary information reported by the diet heart trials. (DOCX 36 kb)

Additional file 2: Funnel plot for major CHD events. (DOCX 27 kb)

Additional file 3: Funnel plot for total CHD events. (DOCX 28 kb)

Additional file 4: Funnel plot for CHD mortality. (DOCX 27 kb)

Additional file 5: Funnel plot for total mortality. (DOCX 27 kb)

## Abbreviations

CHD: Coronary heart disease
CI: Confidence interval
CVD: Cardiovascular disease
DART: Diet and Reinfarction Trial
FMHS: Finnish Mental Hospital Study
HDAT: Houtsmuller Diabetic Angiopathy Trial
LAVAT: Los Angeles Veterans Administration Trial
MCS: Minnesota Coronary Survey
MRCT: Medical Research Council Trial
MUFA: Monounsaturated fatty acids
NDHS: National Diet Heart Study
ODHS: Oslo Diet Heart Study
PUFA: Polyunsaturated fatty acids
RCOT: Rose Corn Oil Trial
RR: Risk ratio
SDHS: Sydney Diet Heart Study
SFA: Saturated fatty acids
STARS: St. Thomas Atherosclerosis Regression Study
TFA: Trans fatty acids
