# Supplementary material for: The effect of replacing saturated fat with mostly n-6 polyunsaturated fat on coronary heart disease: a meta-analysis of randomised controlled trials
Source: Nutr J. 2017 May 19;16:30. doi: 10.1186/s12937-017-0254-5 (PMC5437600; doi:10.1186/s12937-017-0254-5)
Supplement: Supplementary file 1 — The dietary information reported by the diet heart trials. (DOCX 36 kb) [file 12937_2017_254_MOESM1_ESM.docx]

**Additional file 1**

**The dietary information reported by the diet heart trials**

|  | Experimental diet | Control diet |
| --- | --- | --- |
| RCOT | “Patients in both oil groups were instructed to avoid fried foods, fatty meat, sausages, pastry, ice-cream, cheese, cakes (except plain sponge), etc. Milk, eggs, and butter were restricted. An oil supplement of 80 g./day was prescribed, to be taken in three equal doses at meal-times” [1]. | “No advice on dietary fat was give to control patients” [1]. |
| ODHS | “Meat should be restricted as much as possible, and when used, all visible fat should be removed. When boiled meat was eaten, the fat layer following cooling should be removed. Whale beef, a not uncommon meat in Norway, and poultry were recommended as substitutes for beef, mutton, and pork. Fish of all types, and all kinds of shell fish were recommended. Whole milk and cream should be completely eliminated with the exception of one deciliter of milk with the Sunday dessert and to use in coffee. Skim milk was recommended. Butter and fat cheeses should likewise disappear from the diet. Cheeses of a low fat content should be substituted for the usual cheeses rich in fat. One egg with yolk was allowed once a week, and egg yolks occasionally used for baking purposes in the homes were accepted. Otherwise, egg whites could be used in cooking and baking. A loaf made with whole milk and some extra margarine was not to be used. Otherwise, the common types of bread – wheat and rye – were recommended, and the use of brown bread was encouraged. Cereals, such as corn flakes, shredded wheat, and puffed rice, used with skim milk were recommended for breakfast and as a second course at dinner. Porridge made with skim milk or water was to be used. Pure sugar should not be used abundantly. With the exception of coconuts, all foods of vegetable origin, such as salads, beans, peas, cabbage, carrots, fruit, and nuts, were recommended. The average consumption of butter and margarine per head of the Norwegian population per day has in the years 1953-63 been about 12 g and 65 g respectively. When this study started, a typical Norwegian margarine contained only 2-4 per cent of linoleic acid, and the instructions about elimination of saturated fats had to be very strong with regard to margarine, which was entirely restricted. Lard and shortenings were also restricted. Use of olive oil was discouraged. At the start of the trial soy bean oil was the only oil rich in linoleic acid readily available in the market. Abundant use of this oil was recommended, and it should be used for all cooking and baking. As a substitute for margarine and butter, a mixture of skim milk powder and soy bean oil with some salt and coloring material was used. The total amount of dietary soy bean oil desirable was set at half a liter per week. If it proved impossible to use so much in the cooking and baking and in the butter substitute, the daily consumption of 15-30 g soy bean oil as such, taken as a ‘medicine’, was recommended. Alcoholic and non-alcoholic beverages were allowed. To ensure that the recommended diet fulfilled the requirements for vitamins, but mainly as a means of contact, all dieters were given, free of cost, a multiple vitamin preparation of which one tablet was to be taken daily. The controls were also given the same preparation. As a means of contact and stimulation, the dieters twice during the trial also received, free of cost, considerable quantities of Norwegian sardines canned in cod liver oil, which proved to be popular as a bread spread” [2]. | “All dieters were given, free of cost, a multiple vitamin preparation of which one tablet was to be taken daily. The controls were also given the same preparation” [2]. |
| NDHS | Nutrient targets were as follows. Diet B: 30% fat, < 9% SFA, ≥ 15% PUFA, P/S of 1.5. Diet C: 40% fat, < 9% SFA, ≥ 18-20% PUFA, P/S of 2.0. Diet X: 25-30% fat, P/S of 1.0. Diet BC: 30-40% fat, P/S of 1.5-2.0. Diet F: 40% fat, P/S of 3.0. Diet G: 40% fat, P/S of 1.0. Diet Y: 25-30% fat, P/S of 1.0. Methods to meet these targets included the provision of special ‘diet-heart foods’, where the fat was removed and filled with vegetable oils, that were able to be purchased from diet-heart stores to help meet these targets (diet B, C, BC, F and G) and dietary advice to purchase lean meats from the general market and avoid high fat dairy products (diet X, BC and Y) [3]. | Nutrient targets for diet D was 40% fat, 16-18% SFA, 7% PUFA and P/S of 0.4 to reflect average nutrient intakes in the US. Methods to meet these targets included special ‘diet-heart foods’, where the fat was removed and filled with “either animal fat or hydrogenated shortening”, were able to be purchased from diet-heart stores to help meet these targets (first and second study) and by either structured or unstructured dietary advice (second study). Group Z received no dietary advice [3]. |
| MRCT | “Patients on the test diet were instructed to take 3 oz. (85 g.) of soya-bean oil daily”. “At least 43 g. of soya-bean oil daily had to be taken unheated, and it was often drunk with fruit juice. The remainder could be used in cooking”. “Up to 35 g. of other fat per day was also allowed. 14 g. of this was taken as a moderately unsaturated margarine (‘ Blue Band ’). Foods allowed daily included lean meat (up to 85 g.), any fish, skimmed milk, and clear soups. Foods forbidden included butter, other margarines, cooking-fat, other oils, fat meat, whole milk, cheese, egg yolk, and most biscuits and cakes” [4] | “Patients in the control group ate the diet they would ordinarily have taken” [4]. |
| LAVAT | “Saturated fat has been eliminated from the diet in a variety of ways. Butter is rigorously excluded, as are all dairy products which contain butterfat. Conventional margarines and hydrogenated shortenings are omitted except for a few exceptions below” [5]. “The design of the experimental diet involved substitution of vegetable oils for about two-thirds of the animal fat, total fat content being kept around 40%. An attempt was made to stabilize the iodine value of the mixed fat in the control diet at 55 and that in the experimental diet at 100. Multiple vegetables oils were used, including corn, soybean, safflower, and cottonseed, the choice in an individual instance being largely pragmatic”. “Vegetable oils were incorporated into the experimental diet in the form of filled milk, imitation ice cream, "unsaturated" margarine, special sausage products, and filled cheeses. Vegetable oils were used liberally in cooking and baking. Meat fat was minimized by the use of specially trimmed lean cuts” [6]. | “The control diet was a conventional food pattern containing 40% fat calories, mostly of animal origin. It was similar to the regular diet but not identical to it” [6]. |
| FMHS | “The normal diets in use in the hospitals before the trial contained moderate amounts of fats (31-33% of total food energy), over half (54%) of which were dairy fats (mainly from milk and butter). To obtain a good acceptance for the diet, it was decided to change it outwardly as little as possible. The total fat content of the diet was to remain unchanged, and customary foods or modified foods closely resembling them were to be used. The serum-cholesterol-lowering effect was to be obtained through a replacement of the principal dietary fats of the saturated type, dairy fats, by fats of more unsaturated nature. This was done by replacing ordinary milk by an emulsion of soybean oil in skim milk ('filled milk') and by replacing butter and ordinary margarine by a 'soft margarine' with a high content of polyunsaturated fatty acids. The net effect of these changes was that the dairy fats abundantly present in the normal diets were almost totally replaced by vegetable oils, mainly soybean oil. The fat content of the filled milk was identical with that of ordinary milk included in the normal diets, viz 3.9%. The filled milk as well as the soft margarine were fortified with vitamins A and D”. “The comparison of the means of the 2 hospitals shows that the consumption figures were generally very similar during the SCL-diet and normal-diet periods. The only notable unintentional differences were in the consumption of meat and eggs, which were higher during the normal-diet periods”. “A comparison between the means of the SCL diet periods and those of the normal-diet periods revealed a good concordance of the intake figures, except in the case of sucrose which was 16% higher during the normal-diet periods, and of cholesterol in which the difference was intentional” [7]. | “The diet was kept without any intentional change” [8]. |
| SDHS | “Two hundred twenty-one men (the F group) were advised and tutored individually to reduce saturated fat intake to approximately 10% of calories and dietary cholesterol to 300 mg or less per day. They were encouraged to use food containing polyunsaturated fatty acids to 15% or more of daily calories" [9].  “Intervention participants were provided with liquid safflower oil and safflower oil polyunsaturated margarine (“Miracle” brand, Marrickville Margarine). Liquid safflower oil was substituted for animal fats, common margarines and shortenings in cooking oils, salad dressings, baked goods, and other products, and was also taken as a supplement. Safflower oil polyunsaturated margarine was used in place of butter and common margarines” [10]. | “Two hundred thirty-seven men (the P group) were given no specific dietary instruction apart from restriction of calories if thought to be overweight. As a concession to the popular and medical beliefs of the time they were allowed to use polyunsaturated margarine instead of butter if they wished” [9]. |
| HDAT | “Diet II contained carbohydrate 45 cal%, fats 40 cal% (1/3 linoleic acid) and proteins 15 cal%. The linoleic acid content of diet II was 4 times that of diet I, being 20.4 gr/1000 kcal for group II and 5.3 gr/1000 Kcal for group I. The fiber content was similar in both groups as was the cholesterol content, being 88 mg/1000 Kcal in both groups except for 4 patients of group I who preferred butter over saturated margarines. The cholesterol content of the diet for these 4 patients was 148 mg/1000 Kcal” [11]. | “Diet I contained carbohydrate 50 cal%, saturated fats 35 cal%, proteins 15 cal% (diet I)” [11]. |
| MCS | “The treatment diet represented a compromise between the B and C diets of the National Diet-Heart Study, with target values of 45% of calories from fat, a polyunsaturated/ saturated fat (P/S) ratio of 2.5, and less than 150 mg of cholesterol daily” [12].  “The MCE experimental serum cholesterol lowering diet was derived from the “BC” diet of the institutional arm of the National Diet-Heart Feasibility Study at Faribault Hospital. Liquid corn oil was used in place of the usual hospital cooking fats (including hydrogenated oils) and was also added to numerous food items (for example, salad dressings, filled beef (lean ground beef with added oil), filled milk, and filled cheeses). Soft corn oil polyunsaturated margarine was used in place of butter” [13]. | “The control diet involved little departure from the institutional diet served before the trial” [12]. |
| DART | “A reduction in total fat to 30% of energy together with an increase in P/S ratio to 1.0” [14]. “The advice was to use a thin spreading of a polyunsaturated margarine on bread; skimmed milk in all drinks, on cereals and in milk puddings and sauces: chips or roast potatoes no more than once per week and cooked only in a polyunsaturated oil; one shallow-fried item a maximum of once per week and cooked only in a polyunsaturated oil: a maximum of 3 oz high fat cheese and two eggs per week; only lean meats – poultry and white fish – more frequently and lamb or pork no more than once a week. Very lean beef such as topside and silverside was allowed. For dishes requiring minced beef, subjects were instructed to precook the mince and discard the fat before making up the dish. For other major sources of fat (e.g. cakes, pastries, biscuits, meat pies and pasties, crisps, chocolates and toffees) subjects were provided with an exchange list and advised to have a total of no more than four portions of these items per week. Of these, at least two were advised to be made with a polyunsaturated fat to ensure a high P/S ratio. Subjects were advised to replace fat by starch i.e. to eat more bread, boiled and baked potatoes, boiled rice and pasta. A range of low fat alternatives for puddings and snacks were also suggested: e.g. fresh or tinned fruit, jelly, custard or milk pudding made with skimmed milk, mergingue, low fat yoghurt and sorbet. In addition, recipes were provided for other low fat puddings and cakes. Boiled sweets were suggested as an alternative to chocolates and toffees. Canned baked beans and spaghetti were recommended as alternatives for snack meals. Subjects in this group who required weight-reducing advice were advised a much greater restriction of fat-containing foods, to avoid sugar and sugar-containing items and reduce consumption of starch-containing items” [14]. | “Those randomized to the ‘no advice’ group were given a ‘sensible eating’ sheet which did not include advice on any of the intervention dietary components” [14]. |
| STARS | “Total fat intake was reduced to 27% of dietary energy, saturated fatty acid content to 8-10% of dietary energy, and dietary cholesterol to 100 mg/1000 kcal; omega-6 and omega-3 polyunsaturated fatty acids were increased to 8% of dietary energy, and plant-derived soluble fibre (chiefly pectin) intake was increased to the equivalent of 3-6 g polygalacturonate/1000 kcal” [15].  “Following the prescribed dietary guidelines required the subjects to strictly limit their intake of animal protein (eg, meat, cheese, fish), margarine and oils; to avoid processed foods (eg, cookies, pastry, cakes); and to consume relatively large quantities of starchy, low-fat foods (eg, bread, potatoes, pasta) as well as fruits and vegetables (particularly legumes and oats)” [16].  “Overweight patients were prescribed a diet that contained 1000-1200 kcal daily to achieve a BMI of 25 kg/m^2^” [15]. | “The control group received, in common with both intervention groups, cardiological supervision and treatment, repeated counselling against smoking, and antihypertensive treatment if appropriate. All participants were advised about a suitable level of daily exercise. Patients in the control group with a BMI above 25 kg/m^2^ were advised to lose weight but did not receive formal dietary counselling [15]”. |

Abbreviations: RCOT Rose Corn Oil Trial, ODHS Oslo Diet Heart Study, NDHS National Diet Heart Study, MRCT Medical Research Council Trial, LAVAT Los Angeles Veterans Administration Trial, FMHS Finnish Mental Hospital Study, SDHS Sydney Diet Heart Study, HDAT Houtsmuller Diabetic Angiopathy Trial, MCS Minnesota Coronary Survey, DART Diet and Reinfarction Trial, STARS St Thomas Atherosclerosis Regression Study

References

1. Rose G, Thomson W, Williams R. Corn oil in treatment of ischaemic heart disease. Br Med J 1965;1:1531-1533.

2. Leren P. The effect of plasma cholesterol lowering diet in male survivors of myocardial infarction. A controlled clinical trial. Acta Med Scand Suppl 1966;466:1-92.

3. The national diet-heart study final report. Circulation 1968;37:I1-428.

4. Report Of A Research Committee To The Medical Research Council. Controlled trial of soya-bean oil in myocardial infarction. The Lancet 1968;292:693-700.

5. Hiscock E, Dayton S, Pearce M, Hashimoto S. A palatable diet high in unsaturated fat. J Am Diet Assoc 1962;40:427-431.

6. Dayton S, Pearce M, Hashimoto S, Dixon W, Tomiyasu U. A controlled clinical trial of a diet high in unsaturated fat in preventing complications of atherosclerosis. Circulation 1969;40:II-1–II-63.

7. Turpeinen O, Karvonen MJ, Pekkarinen M, Miettinen M, Elosuo R, Paavilainen E. Dietary prevention of coronary heart disease: The finnish mental hospital study. Int J Epidemiol 1979;8:99-118.

8. Turpeinen O, Miettinen M, Karvonen M, Roine P, Pekkarinen M, Lehtosuo EJ, et al. Dietary prevention of coronary heart disease: Long-term experiment. Observations on male subjects. American Journal of Clinical Nutrition 1968;21:255-276.

9. Woodhill J, Palmer A, Leelarthaepin B, McGilchrist C, Blacket R. Low fat, low cholesterol diet in secondary prevention of coronary heart disease. Adv Exp Med Biol 1978;109:317-330.

10. Ramsden CE, Zamora D, Leelarthaepin B, Majchrzak-Hong SF, Faurot KR, Suchindran CM, et al. Use of dietary linoleic acid for secondary prevention of coronary heart disease and death: Evaluation of recovered data from the sydney diet heart study and updated meta-analysis. BMJ 2013;346:

11. Houtsmuller AJ, van Hal-Ferwerda J, Zahn KJ, Henkes HE. Favorable influences of linoleic acid on the progression of diabetic micro- and macroangiopathy in adult onset diabetes mellitus. Prog Lipid Res 1981;20:377-386.

12. Frantz ID Jr, Dawson EA, Ashman PL, Gatewood LC, Bartsch GE, Kuba K, et al. Test of effect of lipid lowering by diet on cardiovascular risk. The minnesota coronary survey. Arteriosclerosis 1989;9:129-135.

13. Ramsden CE, Zamora D, Majchrzak-Hong S, Faurot KR, Broste SK, Frantz RP, et al. Re-evaluation of the traditional diet-heart hypothesis: Analysis of recovered data from minnesota coronary experiment (1968-73). BMJ 2016;353:i1246.

14. Fehily AM, Vaughan-Williams E, Shiels K, Williams AH, Horner M, Bingham G, et al. The effect of dietary advice on nutrient intakes: Evidence from the diet and reinfarction trial (dart). Journal of Human Nutrition and Dietetics 1989;2:225–235.

15. Watts GF, Lewis B, Brunt JN, Lewis ES, Coltart DJ, Smith LD, et al. Effects on coronary artery disease of lipid-lowering diet, or diet plus cholestyramine, in the st thomas' atherosclerosis regression study (stars). Lancet 1992;339:563-569.

16. Watts GF, Jackson P, Burke V, Lewis B. Dietary fatty acids and progression of coronary artery disease in men. Am J Clin Nutr 1996;64:202-209.
