# Supplementary figures and images for: The effect of replacing saturated fat with mostly n-6 polyunsaturated fat on coronary heart disease: a meta-analysis of randomised controlled trials
Source: Nutr J. 2017 May 19;16:30. doi: 10.1186/s12937-017-0254-5 (PMC5437600; doi:10.1186/s12937-017-0254-5)

**Additional file 2**

**Funnel plot for major coronary heart disease events**


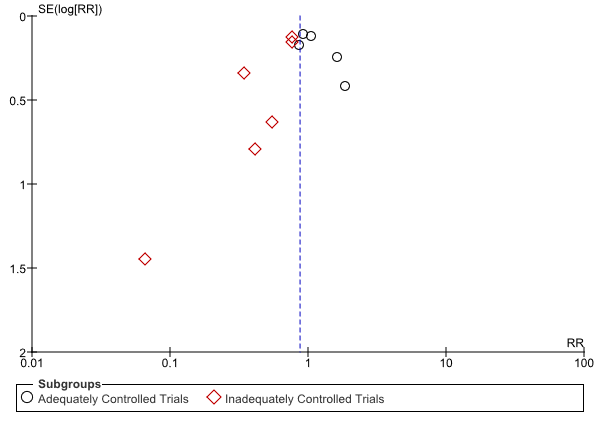

Supplement: Supplementary file 2 — Funnel plot for major CHD events. (DOCX 27 kb) [file 12937_2017_254_MOESM2_ESM.docx]

**Additional file 3**

**Funnel plot for total coronary heart disease events**


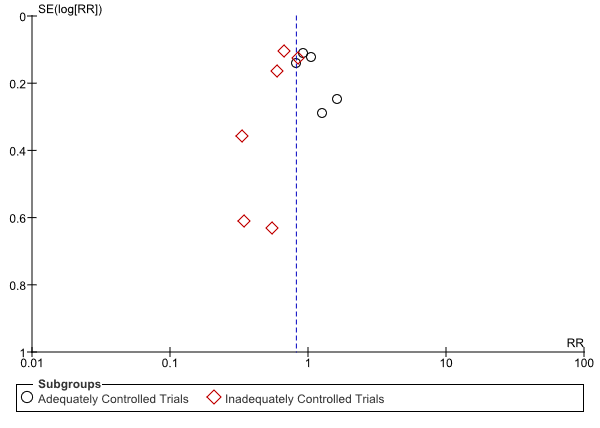

Supplement: Supplementary file 3 — Funnel plot for total CHD events. (DOCX 28 kb) [file 12937_2017_254_MOESM3_ESM.docx]

**Additional file 4**

**Funnel plot for coronary heart disease mortality**


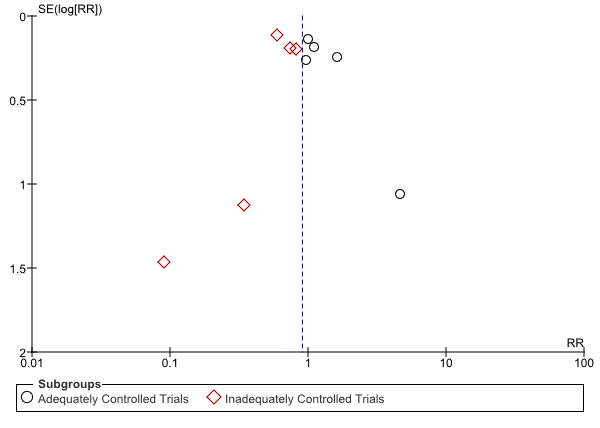

Supplement: Supplementary file 4 — Funnel plot for CHD mortality. (DOCX 27 kb) [file 12937_2017_254_MOESM4_ESM.docx]

**Additional file 5**

**Funnel plot for total mortality**


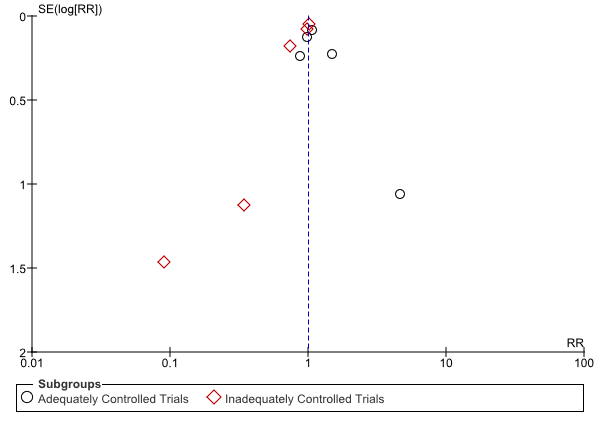

Supplement: Supplementary file 5 — Funnel plot for total mortality. (DOCX 27 kb) [file 12937_2017_254_MOESM5_ESM.docx]
